# Telemetry-controlled simultaneous stimulation-and-recording device (SRD) to study interhemispheric cortical circuits in rat primary somatosensory (SI) cortex

**DOI:** 10.1186/s42490-019-0019-7

**Published:** 2019-08-08

**Authors:** John T. Ramshur, Bashir I. Morshed, Amy L. de Jongh Curry, Robert S. Waters

**Affiliations:** 1 Department of Biomedical Engineering, University of Memphis, 330 Engineering Technology Building, Memphis, TN 38152 USA; 2 Department of Electrical & Computer Engineering, University of Memphis, 204C Engineering Science Building, Memphis, TN 38152 USA; 3 Department of Anatomy and Neurobiology, University of Tennessee Health Science Center, 855 Monroe Avenue, Memphis, TN 38163 USA

**Keywords:** Intracortical microstimulation, Brain-computer interface, Primary somatosensory cortex

## Abstract

**Background:**

A growing need exists for neuroscience platforms that can perform simultaneous chronic recording and stimulation of neural tissue in animal models in a telemetry-controlled fashion with signal processing for analysis of the chronic recording data and external triggering capability. We describe the system design, testing, evaluation, and implementation of a wireless simultaneous stimulation-and-recording device (SRD) for modulating cortical circuits in physiologically identified sites in primary somatosensory (SI) cortex in awake-behaving and freely-moving rats. The SRD was developed using low-cost electronic components and open-source software. The function of the SRD was assessed by bench and in-vivo testing.

**Results:**

The SRD recorded spontaneous spiking and bursting neuronal activity, evoked responses to programmed intracortical microstimulation (ICMS) delivered internally by the SRD, and evoked responses to external peripheral forelimb stimulation.

**Conclusions:**

The SRD is capable of wireless stimulation and recording on a predetermined schedule or can be wirelessly synchronized with external input as would be required in behavioral testing prior to, during, and following ICMS.

## Background

Electrophysiology systems used in neuroscience research to perform recording and/or stimulation in the brain have become increasingly smaller, more power efficient, and completely implantable. Some implanted systems operate as tethered devices requiring a cabled connection between the device and computer system [1]. Tethered systems have the advantage of uninterruptible power and higher data bandwidth, but can limit an animal’s range of motion. Whereas wireless systems are less intrusive and extend the range of motion, they nonetheless often require larger and more complex designs with shorter operating times, and less data bandwidth [2–5].

A growing need exists for neuroscience platforms that can perform simultaneous chronic recording and stimulation of neural tissue in a wireless fashion together with signal processing for analysis of the chronic recording data. Intracortical microstimulation (ICMS) has been used for mapping the motor cortex [6, 7], examining sensory input and motor output relationships in motor cortex [8], studying sensorimotor [9] and thalamocortical connectivity [10, 11], and characterizing cortical plasticity [12]. Chronic or repetitive ICMS has been shown to enhance neuronal firing rates [13], increase efficiency of transcallosal pathways [14], and induce long-term potentiation within primary somatosensory (SI) cortex in brain slice preparations [15] and in anesthetized rats [13, 16]. We reported that repetitive stimulation of layer V neurons in the forepaw barrel subfield in rat SI cortex strengthens interhemispheric connections between homotopic sites and leads to expression of previous ineffective ipsilateral input in anesthetized rats [17, 18]. Our long-term objectives are to determine the time-course and behavioral effects of these newly expressed inputs in SI cortex in freely-moving and awake-behaving animals.

System specifications in the present study require that the stimulation-and-recording device (SRD) dimensions (size and weight) be wearable by a rat, capability for wireless simultaneous stimulation and recording in different brain regions, and synchronization with external stimuli. Commercially available implantable devices for use in rodents with all these capabilities exist but are far more costly than the SRD described in the present study. Existing systems used in research are capable of wirelessly transmitting recorded neural signals [2, 4, 19, 20] and wirelessly controlling stimulation [21], but some systems are limited by the use of noncommercial ASIC designs [2, 4, 21, 22] or do not provide on-board synchronized recording with external stimuli [5, 23, 24] but this is not without exception [21]. Devices capable of simultaneous surface electrical or optical stimulation and recording have also been developed with low-power consumption and high noise immunity [22, 25, 26].

Here, we describe the development, design and testing of a wireless SRD in anesthetized and awake, freely-moving rats. We previously reported preliminary stages of our SRD design [17], which follows Ye and colleauges [5]. Additionally, the SRD is low-cost, uses commercial off-the-shelf components and open-source software, provides the capability to simultaneously record and deliver ICMS in multiple brain regions within the depths of the cortex, and synchronizes recording and/or stimulation with external stimuli.

## Results

### Bench testing

Power consumption was evaluated by measuring input current to the microcontroller unit (MCU) and for each subsystem and/or primary components of the subsystem during normal operating conditions including stimulation, recording, and wireless transmission. The summed averaged current required by SRD components, excluding voltage converting circuits, was approximately 23.9 mA with a power consumption of 92.2 mW while stimulating from a single channel (biphasic, ±255 μA, 1-ms pulse duration, 1 Hz) and recording from 12 channels (sampling and transmitting 100-ms traces/s) (Table 1). Including voltage converting circuits, total measured input current averaged over 15 s of operation was approximately 27 mA. During continuous biphasic stimulation (±100 μA, 1-ms pulse duration, 1 Hz, single channel) and recording traces (transmitting 50 (100-ms) every 3 min, single channel), the operating time of the SRD from full charge of the single-cell lithium polymer (LiPo) battery to an operating voltage of 3.45 V was approximately 37 h.

**Table 1:** Average current and power consumption

Subsystem	Component(s)	Current (mA)	Power (mW)
Stimulation	AMUX + Mirrors	1.0	10.0
MCU	PSoC (w/ LED)	8.2	27.1
Wireless	BT (w/ LED)	13.8	52.1
Recording	Intan RHD2216	0.9	3.0

*AMUX* Analog multiplexer, *LED* Light-emitting diode, *BT* Bluetooth

**Fig. 1:**
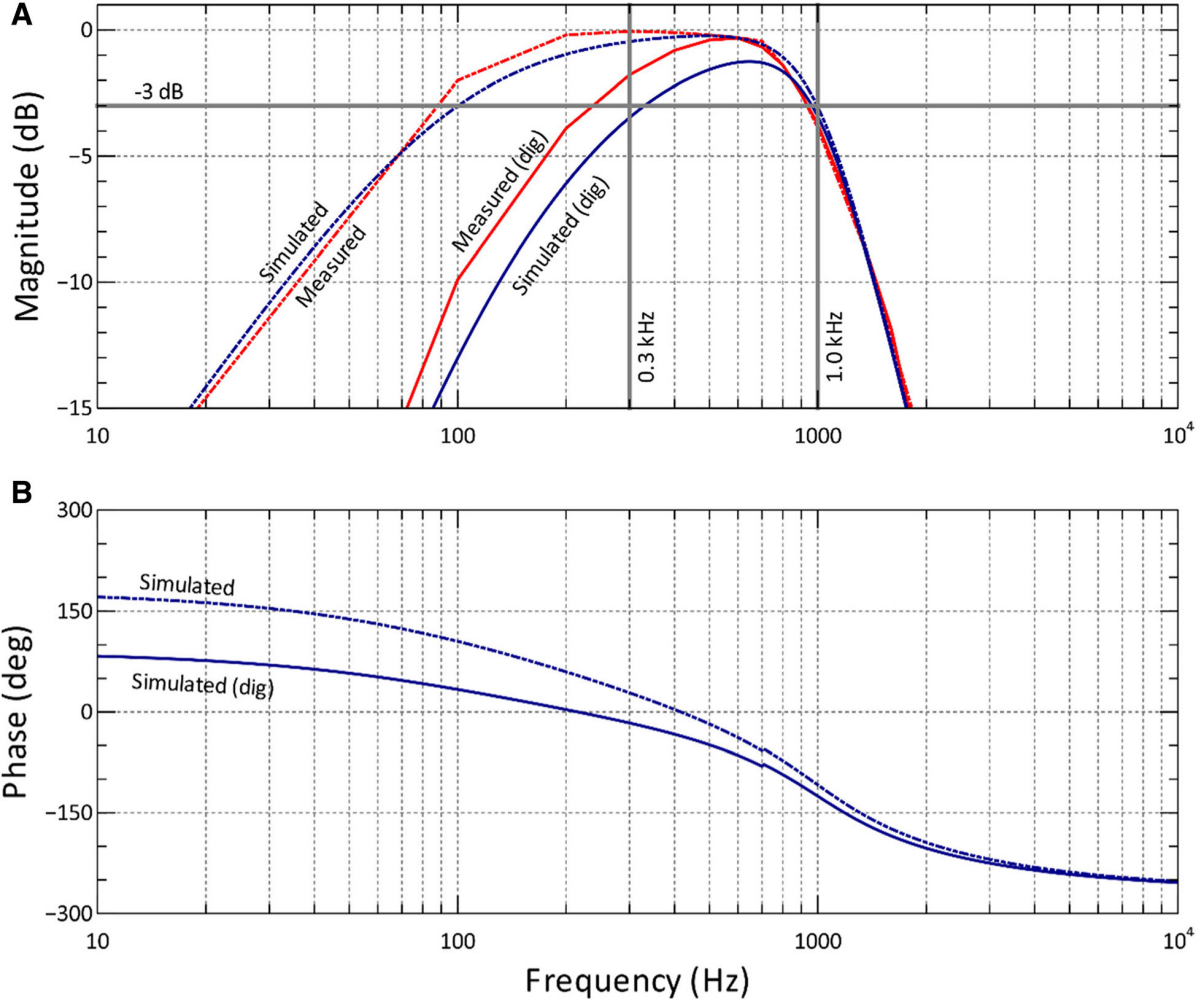
**a** Frequency response of simulated (blue) and measured (red) gains for f_L_ = 100 Hz and f_H_ = 1 kHz. Vertical lines represent the desired overall filter bandwidth of 300 Hz − 1 kHz. The horizontal line marks − 3 dB gain. **b** Frequency response of simulated phase angle. Dashed curves represent frequency responses with the digital high-pass filter (offset removal filter) disabled, and solid curves represent the digital filter–enabled f_D_ = 318 Hz. digital: dig

Frequency response plots of simulated gain, gain computed with bench measurements, and frequency response plots of the simulated phase are shown in Fig. 1. All plots were produced with analog filter bandwidths set to 100 Hz–1000 Hz and the optional digital high-pass filter either disabled or set to 318 Hz. With no digital filter enabled, both the simulated and bench results were comparable. With the digital filter enabled, the lower cutoff frequency (f_L_ = 300 Hz) at − 3 dB was 89 Hz less in bench test versus simulated results. Upper cutoff frequency (f_H_ = 1 kHz) was comparable for both bench and simulated results. Measured f_L_ was not ideal relative to the desired 300 Hz but could be shifted closer to 300 Hz by choosing a higher analog f_L_ or higher digital high-pass filter frequency (f_D_).

### In-vivo testing

**Fig. 2:**
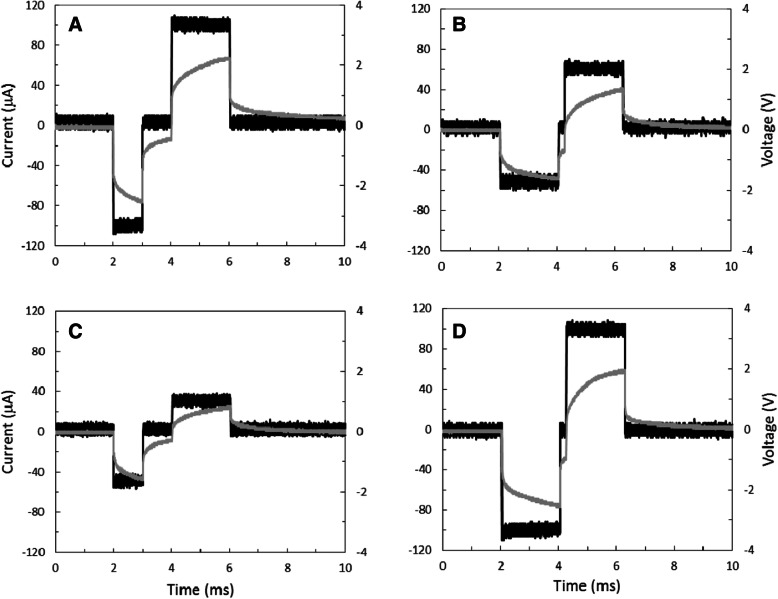
In-vivo examples of pseudophasic (non-symmetric biphasic) (**a**, **c**) and symmetric biphasic (**b**, **d**) constant current stimuli delivered to rat SI cortex with 100-kΩ platinum-iridium (Pt/Ir) microelectrode. Measured output current (black line) and simultaneously measured voltage drop (gray line) across the tissue/electrode load are shown: **a** cathodic: 100 μA, 1 ms; anodic: 100 μA, 2 ms; **b** cathodic: 50 μA, 2 ms; anodic: 60 μA, 2 ms; **c** cathodic: 30 μA, 1-ms; anodic: 50 μA, 2 ms, and **d** cathodic/anodic: 100 μA, 2 ms. Inter-pulse intervals are 1 ms (**a**, **c**) and 0.2 ms (**b**, **d**)

Stimulation testing for multiple waveforms included monophasic, biphasic, and pseudophasic current pulses. Four stimulation current waveforms delivered to layer V of the rat SI cortex by the SRD using various amplitude and timing settings are shown in Fig. 2. Based on these stimulator tests, the maximum compliance voltage required to generate 100 μA using a 100-kΩ electrode was 2.6 V and well below the compliance voltage of 10 V of the SRD. Leakage current measured from the SRD during interphase intervals was 9 nA.

**Fig. 3:**
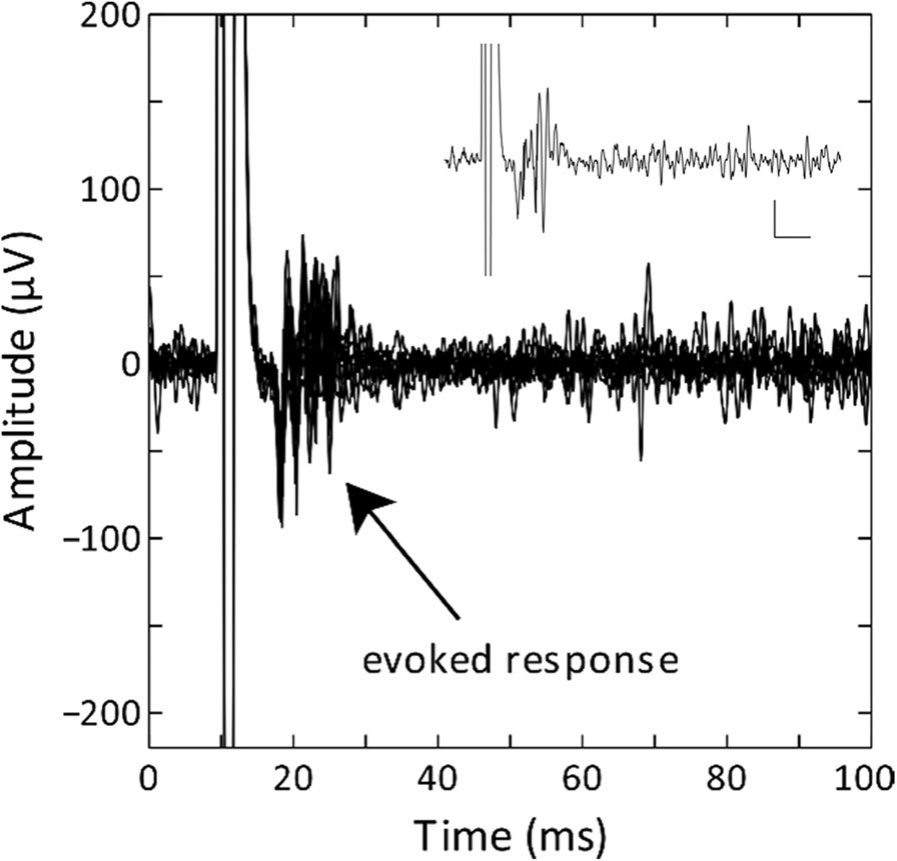
A 1-MΩ microelectrode was implanted in SI in the wrist/forearm representation (right hemisphere) and was used to record evoked responses to stimulation of the contralateral wrist/forearm. A total of 10 evoked responses were recorded during peripheral stimulation (100 μA) of the contralateral forelimb on day 21. Inset: Single trace recorded at the same cortical location. Legend axes represent 30 μV and 10 ms

Electrodes were implanted in the SI wrist representation of one rat for 15 days and in an overlapping wrist/forearm representation in a second rat for 49 days. Spontaneous activity and contralateral forelimb input were recorded in each hemisphere. Peripheral forelimb stimulation was delivered from a constant current stimulus isolation unit (NeuroData) and synched to the infrared (IR) detector that triggered the SRD to collect evoked response recordings. Recordings were measured on post-implant days 1, 2, 3, 6, and 8 in one rat and on post-implant days 6, 8, 21, and 48 in the second rat. Post-implant measured receptive fields were found to be similar to pre-implant receptive fields. Spontaneous neuronal firing and contralateral forelimb evoked responses recorded in wrist/forearm representation recorded at 21 days post-implant are shown in Fig. 3.

**Fig. 4:**
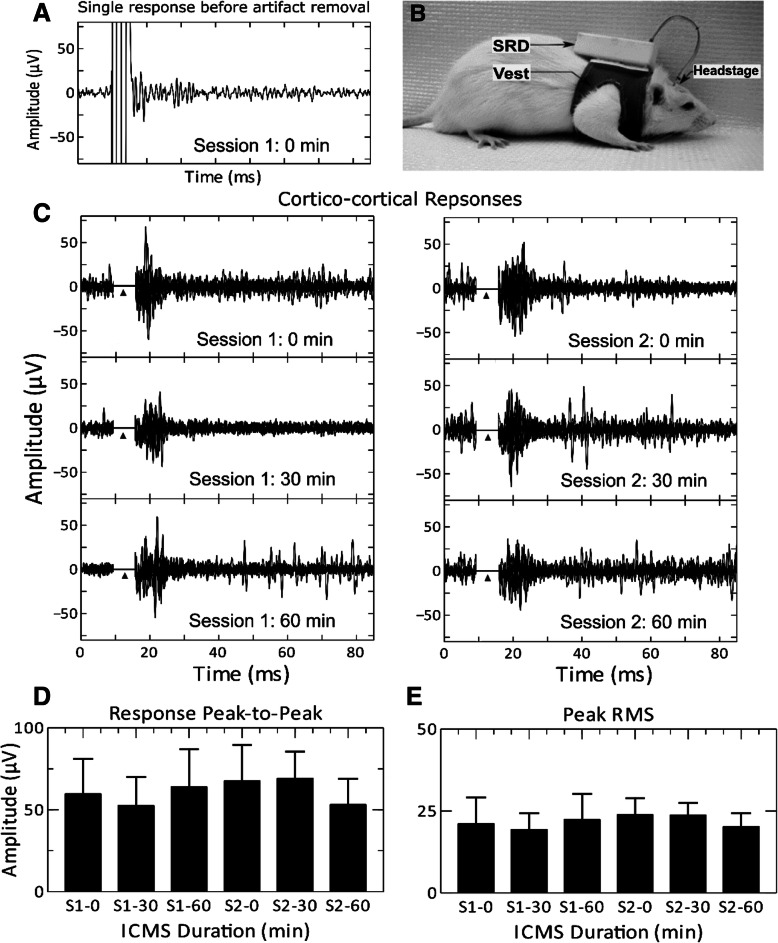
**a** Cortico-cortical evoked response to ICMS delivered to the wrist representation at 8 days post-implantation. **b** Photograph of rat wearing the SRD system. The SRD was fixed to the vest with Velcro and connected to the electrode interface board (EIB) on the headstage using a wire interconnect. Cortico-cortical evoked responses to ICMS delivered to the wrist representation at 8 days post-implantation are shown in (**c**). Each of the six rows of traces represents cortico-cortical evoked responses seen during two 60-min sessions of chronic ICMS (100 μA). Each plot shows 10 traces. Triangle (▲) indicates location of stimulus artifact which was removed after data was transmitted to the host PC. Artifact was replaced with a value of zero if rectified trace sample was greater than the maximum rectified amplitude of the post-stimulus evoked response. **d** Mean peak-to-peak signal amplitudes for each time point in (**b**) are shown with error bars representing one standard deviation. **e** Mean instantaneous RMS peaks for each time point in (**b**) are shown with error bars representing one standard deviation

An example of SRD-controlled simultaneous recording and stimulation record in a freely moving rat is shown in Fig. 4. The photograph (Fig. 4b) shows a rat wearing the SRD and implanted headstage. In this rat, tested on post-implant day 8, two-60 min sessions of chronic ICMS were administered (Fig. 4c). Stimulating and recording electrodes were implanted in homotopic sites within the wrist representation in layer V of SI cortex. Cortico-cortical evoked response amplitudes ranged from 59.8 ± 21.3 μV at the start of session 1 to 53.2 ± 15.6 μV at the end of session 2 and these results are shown in Fig. 4d. Peak instantaneous root mean square (RMS) amplitudes (Fig. 4e), ranging from 21.1 ± 8.0 μV at the start of session 1 to 20.3 ± 4.1 μV at the end of session 2, were not statistically different across sessions (*p* = 0.10). It should be noted that the stimulus pulse duration (1 ms) and amplitude (100 μA) values were geared for interhemispheric stimulation and response, and thus very likely produced the upper limits for stimulus artifact duration in the evoked responses.

## Discussion

In the present study, the SRD, under telemetry-control, was shown capable of simultaneously delivering ICMS for extended periods (tested up to 7 weeks) and recording evoked responses and spontaneous neuronal responses at homotopic sites in the opposite forelimb representation of SI cortex in unanesthetized, freely moving rats. During each postsurgical day of experimentation, we successfully recorded spontaneous cellular spike and bursting activity. We recorded evoked responses in SI cortex synchronized to contralateral peripheral forelimb stimulation and synchronized to ICMS in the opposite SI cortex. Receptive fields were similar before implantation, immediately after implantation, at post-recovery days, and immediately prior to euthanasia in all rats studied.

To minimize the stimulus artifact as seen in our cortico-cortical evoked responses, a biphasic pulse with shorter duration cathodic phase (0.2 ms) followed by 0.1-ms anodic phase would be used; we and others have used this cancellation technique in mapping connectivity between sensory and motor cortices [8, 9]. The large ceiling cortico-cortical stimulation current, used in the present study, can similarly be lowered to reduce stimulus artifact to allow ICMS and recording.

Repetitive stimulation and recording may lead to damage from infection, inflammation, and scarring around electrode sites. Glial scar formation or gliosis and neuronal cell viability around the electrode could be studied using immunohistochemistry [27, 28] or by monitoring total impedance of the electrode-tissue load because gliosis increases impedance [29] and reduces neuronal spike amplitudes [28]. A standardized method of impedance measurement using the electrophysiology interface chip could be employed to allow in-vivo monitoring of total impedance throughout animal survival. Moreover, the ability to adjust electrode depths could circumvent scarring problems due to gliosis around the electrode tip [30]. Wearable microdrive systems are commercially available (Microprobes, EDDS Microdrive System; Cambridge NeuroTech, Nano-Drive). Electrodes could be implanted at a shallow depth and subsequently lowered to a desired depth. Another option would be to independently drive the electrodes postrecovery for fine tuning of position.

### Future design considerations

Several options exist to reduce power consumption of the hardware and firmware, including power-gating unused portions of the circuit and optimizing the wireless configuration and the programmable system-on-chip (PSoC) sleep and awake states. All chips on the SRD have enable/disable pins that can further regulate power consumption. Disabling an entire power domain would eliminate wasted power due to a component’s quiescent currents (analog circuits) and static/leakage current (digital circuits). Power could be further conserved by placing the PSoC into lower power operating modes, such as sleep mode, more often than in the current firmware implementation. Additionally, a combination of data compression, local data buffering, and transmission of only events such spiking activity could be implemented to reduce power consumption.

In the SRD configuration presented here, the system allows for two simultaneous recording channels. This limitation is defined by data transmission speeds, transmission power requirements, MCU speed, and data buffer size. Increasing any of these would allow increased ability to record from more channels simultaneously. The MCU chip used in the SRD is sufficient for our application; however, for more data throughput or processing power, the PSoC 3 may be replaced with a PSoC 5 Low Power (LP) chip with 256 KB Flash and 64 KB SRAM verses the PSoC 3’s 64 KB Flash and 8 KB SRAM. The increased SRAM of the PSoC 5 LP would allow 8 times more signal data buffering if using the SRD’s current buffering scheme, and allow 16 channels to be simultaneously buffered instead of two. At this time, Bluetooth data speeds remain a limiting factor, but buffered data could be transmitted two channels at a time. The footprint of the PSoC 5 LP matches the SRD’s printed circuit board (PCB) layout, and the power and general purpose I/O pins are located at the same pins. Drawbacks to using the PSoC 5 LP would be a potential increase in power usage, and development effort to port the firmware code to a different processor.

Configuration improvements to the SRD electrophysiology interface chip could reduce the stimulus artifact. The chip is currently used in a monopolar, rather than bipolar, configuration. An improved configuration would connect all negative inputs to a common point on the animal via a separate reference bone screw in the skull distal to the ground screw. Due to the potentially long time-constant associated with f_L_, the fast settle function could be turned on just before stimulation occurs, then promptly turned off following the end of the stimulation pulse.

The size of the SRD’s PCB was based on the size of the battery, and thus the size of the PCB was not optimized. However, if a smaller battery is used due to shorter operating time requirements or reduced power consumption, a smaller PCB could be designed to reduce weight and size of the SRD. A new digital electrophysiology interface chip with on-chip stimulation capabilities (Intan Technologies) is now available. With a chip combining amplifiers, analog-to-digital converter (ADC), and stimulation, the stimulator circuit and stimulator power supply could be eliminated, leading to a significantly smaller PCB, which could be designed with the head stage and mounted together as a single unit on the skull. This could also reduce stimulus artifact by eliminating transfer of analog signals along the interconnecting wires that can introduce both electromagnetic coupling and motion artifact.

As currently designed, the head stage was attached to the SRD by an external cable that ran from the head to the dorsally-mounted SRD. Although, we did not observe the cable interfering with either stimulation, recording or animal movement, a future design could run the cable leads subdermally to the SRD or miniaturize the SRD to mount directly on the skull.

## Conclusion

We described the system design, development, testing, and implementation of a wireless SRD to study cortical circuits in an awake rat animal model. No research or commercial system existed that met our experimental requirements for simultaneous stimulation and recording and wireless synchronization from an external input. The SRD successfully functioned in freely moving rats with chronically implanted electrodes up to 7 weeks. Low cost, open-source platform, and wide recording bandwidths make the SRD an attractive system for many electrophysiological applications.

## Methods

**Fig. 5:**
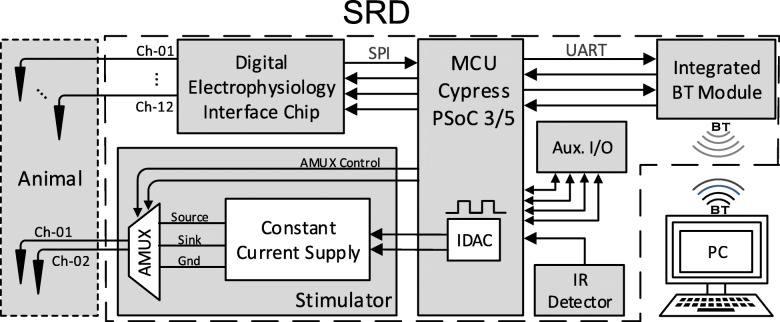
SRD system overview showing interconnections between the microcontroller unit (MCU) with on-board current-mode digital-to-analog converters (IDAC), universal asynchronous receiver/transmitter (UART), serial peripheral interface (SPI), Bluetooth (BT) module, host PC, stimulator with analog multiplexor (AMUX), digital electrophysiology interface chip (recorder), auxiliary input/output (Aux. I/O), infrared (IR) detector, and electrodes (e.g. Ch-01, Ch-02) within animal tissue. Figure modified from [21].© 2014 IEEE. Reprinted, with permission, from IEEE Proceedings

An overview of the SRD subsystems and component interconnections is shown in Fig. 5. Biopotential signals from biological tissue are amplified, filtered, and digitized by an integrated digital electrophysiology interface chip. Digitized data are transferred to the MCU via serial peripheral interface (SPI) communication to a core processor and then buffered and transmitted to a host personal computer (PC) via BT for visualization and offline analysis. BT communication is used to send stimulation, recording, calibration, and filter settings to the SRD from a graphical user interface (GUI). Biological tissue stimulation is delivered by an adjustable constant current stimulator capable of ±255 μA of current with a compliance voltage up to ±10 V.

The MCU is a PSoC with 8051 microprocessor (PSoC® 3, version CY8C3866, Cypress Semiconductor). The PSoC manages all aspects within the SRD including timing, analog sampling, stimulus control, auxiliary input/output (I/O), external IR sensing, and wireless communications. The PSoC package chosen was an 8 mm × 8 mm, 68-pin quad-flat no lead package. Additional SRD features are a low-profile zero-insertion force (ZIF) socket to allow reprogramming of the PSoC, a second ZIF header for auxiliary analog or digital I/O, a button for system-wide reset, a tri-color (red-green-blue) light-emitting diode (LED) for status indications, and an IR sensor for external triggering. The auxiliary header includes a semi-isolated and decoupled range of pins for improved noise immunity.

### Recording subsystem

Analog biological signals are digitized using an electrophysiology interface chip (RHD2216, Intan Technologies) with programmable analog and digital filters, 16-bit ADC, 30 ksps/channel sample rate, bipolar or unipolar configurations, in-situ electrode impedance measurement capability, and 16-bit SPI communication. The SRD was configured for 12 unipolar recording channels with a maximum sample rate of 15 ksps/channel. Users can select any two channels from the 12 available, or sweep through all channels two at a time for the programmed trace length. Sample rate, filter settings, and channel selection are adjustable using the custom GUI. An18-pin keyed connector (A79043, Omnetics) was used as the analog I/O header for cortical signals from electrodes and stimulation waveforms to the cortex.

Upper and lower bandwidths of the amplifiers can be dynamically programmed by means of internal registers on each chip allowing optimization for different types of electrophysiological signals including electrocardiogram, electromyogram, electrocorticogram, electroencephalogram, neural spikes, and local field potentials. A third-order Butterworth low-pass filter defines the upper cutoff frequency (f_H_) and is adjustable from 100 Hz to 20 kHz. Lower bandwidth frequencies (f_L_) are defined by a first-order high-pass filter and are adjustable from 0.1 Hz to 500 Hz. An optional digital offset removal feature can be enabled within the GUI to remove the residual DC offset voltages associated with the analog amplifiers. The digital cutoff frequency (f_D_) is automatically computed within the GUI based on the sampling rate and is selected by the user from a drop-down menu of f_D_-values available for the chosen sampling rate.

To characterize the SRD filters, both simulations and physical measurements were performed. Gain and phase calculations were computed in MATLAB using f_L_ = 100 Hz and f_H_ = 1 kHz, and with f_D_ enabled and set to 318 Hz. Frequency responses were measured using the same filter bandwidths and digital filter options as above and sweeping a sine wave with known amplitude through a range of frequencies. At each frequency step, input magnitude, sampled output magnitude, and frequency were recorded. Similar studies often use low-pass filter values of 3–5 kHz or higher and include spike sorting in off-line analysis. Our chosen bandwidth is typical for extracellular recordings performed in our laboratory to reduce high frequency noise [9, 17, 18, 31, 32] and allows for post-processing analyses to characterize the spiking activity of evoked responses such as firing rate, duration and latency.

### Stimulating subsystem

A constant current stimulation circuit allows delivery of monophasic, biphasic, or pseudophasic current pulses. Current pulses can be directed to one of two monopolar electrodes. The PSoC’s on-board current-mode digital-to-analog converters (IDAC) have 3 output ranges (2,040 μA, 255 μA, and 31.875 μA) and two polarities (sink or source). We selected the 255 μA range, which gives 1-μA increments. To produce a current of desired specifications, IDAC outputs were conditioned using a three-stage process.

The first stage uses two built-in PSoC IDACs to produce one 0–255 μA current source and one 0–255 μA current sink; each has a compliance voltage of around 1 V. Compliance voltage is defined as the voltage required to maintain constant current across the combined animal and electrode load. To boost the compliance voltage, the second stage comprises two current mirrors, each constructed using one dual N-channel and dual P-channel matched metal-oxide-semiconductor field-effect transistor (MOSFET) (ALD1105, Advanced Linear Devices). Compliance voltage of the current sink mirror and current source mirror is − 10 V and + 10 V, respectively. The third stage directs current into one of two available electrodes for tissue stimulation or shorts both electrodes to ground for discharging. Analog switching used a precision low-voltage analog multiplexer (DG9409, Vishay) that includes break-before-make action, low on-resistance (3.9 Ω), fast on/off switching times (4.2/24 ns), and a simple two-wire logic interface. Switching decisions are controlled by two general-purpose I/O pins on the PSoC. With an on-resistance of 3.9 Ω, the resulting additional voltage drop across the device during stimulation is negligible (~ 1 mV).

### Wireless communication subsystem

The SRD is capable of bidirectional wireless communication using a BT 2.1 module PCB antenna (RN42 Class 2, Microchip). The BT port was configured in the serial port profile with universal asynchronous receiver/transmitter setting of 115,200 bps baud rate. A red status LED on the SRD visually indicates the module’s operating mode including command mode (blink 10×/s), configurable mode (blink 2×/s), discoverable/idle mode (blink 1×/s), and connected mode (solid on).

The SRD uses two framing bits (1 start bit and 1 stop bit) per byte of data acquired. The maximum amount of data required to transmit one trace is less than 50 kbits when sampling two channels at a rate of 15 ksps and a trace length of 100 ms.

### Printed circuit board

**Fig. 6:**
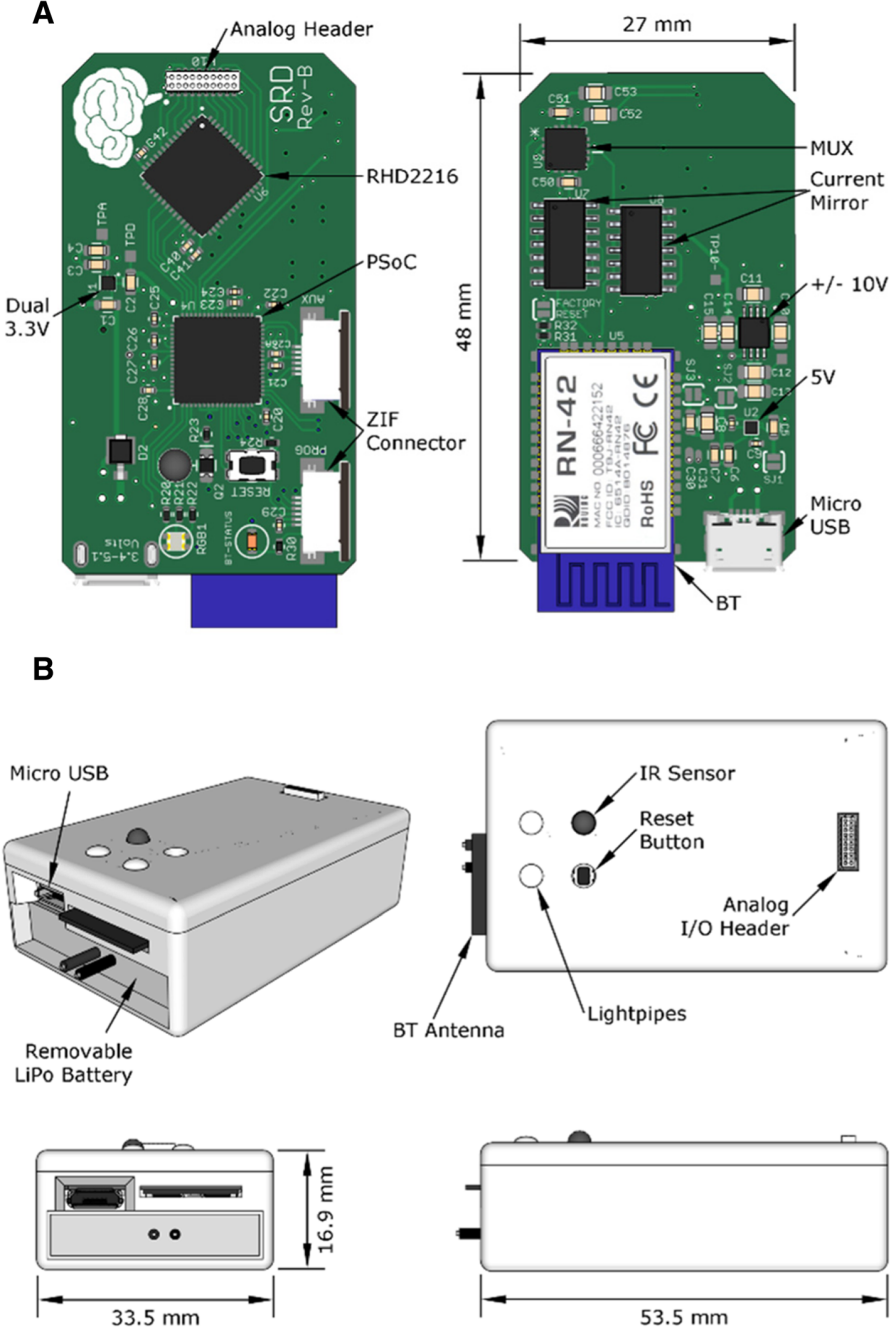
**a** PCB component layout of top and bottom sides of the SRD. **b** 3D model of SRD enclosure with several key features highlighted including the exposed micro USB connector, removable battery, exposed BT antenna, light pipes, IR sensor, recessed reset button, and analog header. The enclosure lid is held onto the primary enclosure body by four pair of 2 mm × 2 mm cylindrical neodymium magnets

The PCB was designed with Eagle CAD v6 (CadSoft) and constructed using a 1.6-mm 4-layer PCB with FR-4 material (Pentalogix) (Fig. 6a). Top and bottom copper layers contain signal layers with no copper fill, the second copper layer contains a ground plane, and the third copper layer contains a digital and analog power plane. BT module, stimulator power supplies, and stimulator circuits are located on the bottom layer to distance the potentially noisier elements away from the more sensitive analog components on top. The top layer is separated into a region for analog routing around the electrophysiology interface chip and a region for digital routing around the PSoC. All electrophysiology signals are routed to/from the electrophysiology interface chip via a small analog header (A7518–801 male connector, Omnetics) located near the edge of the PCB. Analog signal trace lengths were kept as short as possible between chip and header. The digital partition of the top layer contains all LEDs, programming connector, auxiliary connector, reset button, and PSoC. The analog and digital dual low-dropout (LDO) regulator was placed between the two partitions to minimize voltage drop.

**Fig. 7:**
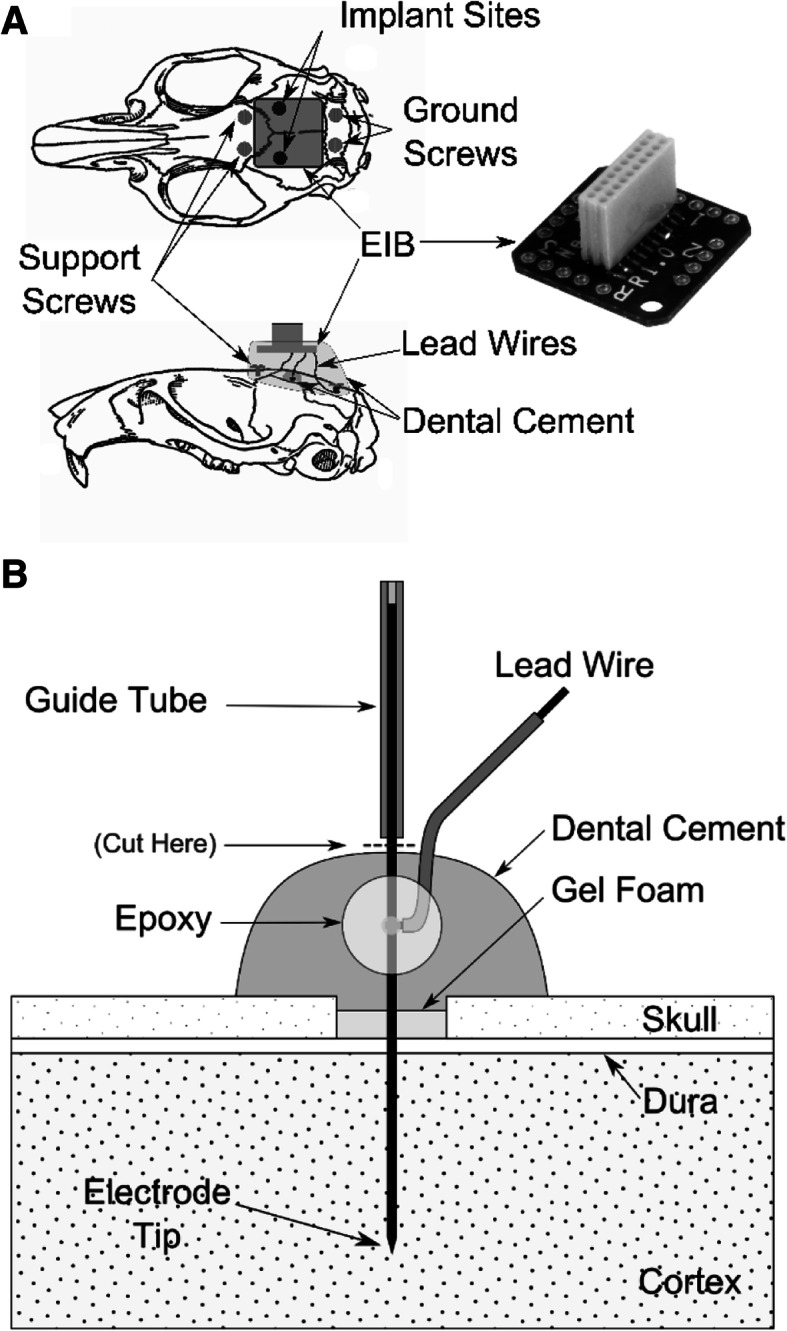
**a** Illustration of SRD headstage with EIB, approximate locations of implant sites, screws, lead wires, and encapsulating dental cement. **b** Illustration of chronic electrode implanted into cortex. Electrode is secured to the skull by dental cement. After dental cement was cured, the remaining electrode shaft and guide tube were cut just above the cured cement

To stimulate and record in an awake animal for extended periods of time, the SRD is wearable and connected to a headstage affixed to the animal’s skull. A custom enclosure was designed to fit tightly around the PCB with 1 mm clearance (Fig. 6b). The SRD enclosure was attached via Velcro to a stretchable rodent jacket (Lomir Biomedical). The headstage consists of a chronically implanted stimulating electrode, implanted recording electrode, and an electrode interface board (EIB) [EIB-16, Neuralynx, Inc.] attached to the rat’s skull (Fig. 7). Headstage and SRD are connected via a detachable and flexible wire interconnect.

### Power supply subsystem

Power to the SRD is derived from a rechargeable single-cell LiPo battery, which can be recharged with any voltage input from 3.5–5.1 V via a micro-USB jack. The 850 mAh LiPo battery has a protection circuit attached to prevent damaging discharge or recharge. The SRD uses four separate power domains to power analog, digital, negative stimulation, and positive stimulation circuits. Digital and analog power domains each use a separate 3.3 V rail and are generated using one dual LDO linear regulator (MIC5393, Micrel). Drop out voltage is less than 155 mV at 150 mA, which allows the SRD to operate until the battery drains to near 3.45 V. Stimulator power is generated in two stages. Stage one boosts the LiPo battery voltage to 5 V using a 30-mA inductor-less boost converter (AS1302, AMS). Stage two uses a dual-output charge pump (MAX865, Maxim) to convert 5 V into − 10 V and + 10 V rails. To evaluate power consumption, input current was measured for each subsystem and/or primary components of the subsystem during normal operating conditions including stimulation, recording, and wireless transmission. Currents were measured by a precision current meter (μCurrent Gold, EEVBlog) and sampled at 10 ksps (DI-155, DATAQ Instruments). Cumulative current consumed by stimulation components (analog multiplexer, or AMUX, and current mirrors) was measured while generating biphasic stimulus pulses (±225 μA, 1-ms pulse, 1 Hz). Current and power consumption were averaged over 15 s of sampled data. The wireless components including the BT module and indicator LED were tested when operating with sniff mode set to 100 ms and transmitting 100-ms traces every 1 s. Average current consumption of the recording subsystem was measured while 12 channels were activated and sampling 100-ms duration traces every 1 s at 15 ksps.

A battery discharge test was performed using the SRD as the load, simulating a prolonged awake animal experiment. The SRD was set to operate in automatic mode with biphasic stimulation (±100 μA, 1-ms pulse, 1 Hz) and fifty 100-ms traces transmitted to the PC every 3 min. Battery voltages and output currents were monitored and logged using a custom Arduino-controlled battery test station that disconnected the load from the battery when the voltage drained to 3 V.

### IR detector and external IR sync pulse emitter

Applications often require the user to sync the SRD recording or stimulation with an external system. We use an external device to deliver peripheral stimulation while simultaneously recording evoked cortical responses. Behavioral responses such as activating a bar-press could be used to deliver ICMS while recording cortical responses. To adhere to the overall wireless design of the SRD, an IR-based solution was chosen. To accommodate an external IR trigger (e.g. bar press by rat causing external IR trigger pulse to be emitted), the SRD includes an onboard IR sensing circuit capable of triggering a recording or stimulation event. The IR pulse sensor is an IR sensitive phototransistor (MTD8000M3B, Osram) connected to a PSoC I/O pin configured as a digital input using the resistive pull-up mode.

A separate PCB was designed and manufactured that allows three 5-V pulses from external devices to generate IR sync pulses, as input to the SRD’s IR pulse detector. Power for the IR pulse emitter board is provided using a single-cell LiPo battery or three standard AA batteries and 3.3 V LDO linear regulator (LD3915, STMicroelectronics). Incoming 5-V square wave pulses cause an N-channel−enhanced mode MOSFET (PMV40UN, NXP Semiconductors) to allow current flow through a high-intensity IR emitter (SFH 4235, Osram). Emitted IR light pulses are of the same duration as the original input pulse, or less than 10 ms to prevent damage to the IR emitter. A standard LED is used to give a visible confirmation of IR pulses. A monostable circuit (TS555, STMicroelectronics) is used to create a visible light pulse with duration independent of the input or IR pulse duration. The PCB is housed inside a clear-top water-resistant enclosure with on/off toggle switch and input signal connector.

Total SRD latency, from start of trigger signal to initiation of data sampling by the SRD when operating in external recording mode, is approximately 18 ms after the external IR device initiates an IR pulse at a distance of 12 in. For combinations of distances (6 in. to 24 in.) and angles (30° to 60° relative to horizontal), pin latencies with ambient lights turned on are shorter (5–50 μs) compared to ambient lights turned off (12–170 μs). At 12 in. and angles of 30° and 60° relative to horizontal, latencies increase by less than 8 μs with ambient lights on and less than 24 μs with ambient lights off.

### Software design

**Fig. 8:**
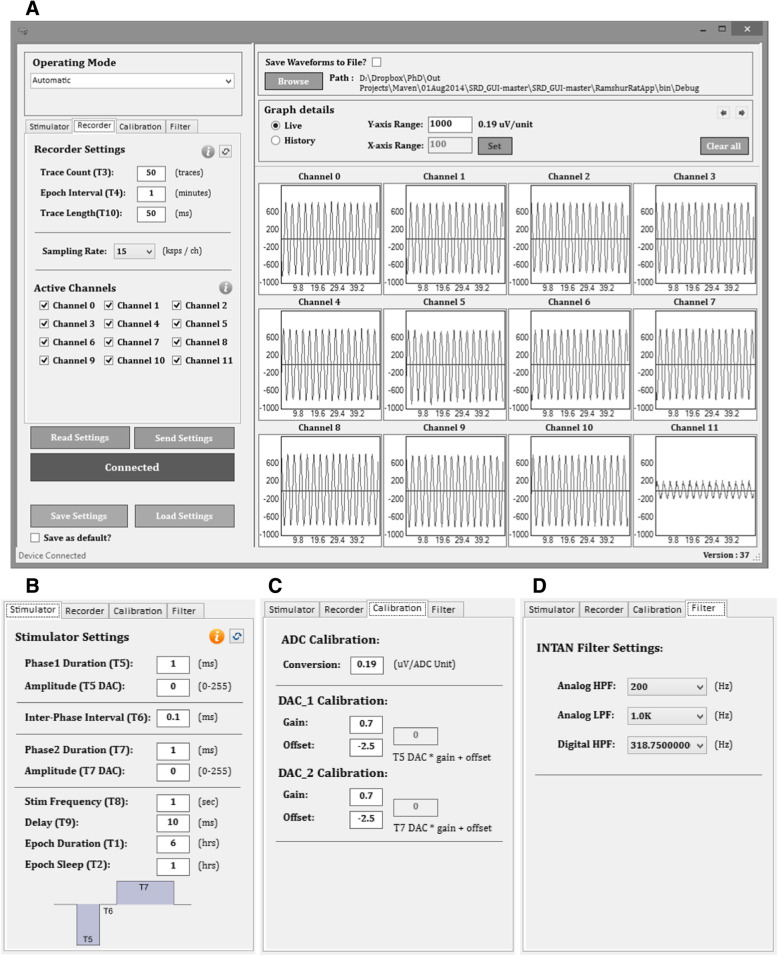
**a** Screenshot of the GUI showing live streaming of a sine wave to all SRD input channels with the Recorder settings tab selected. The sections of the GUI are operating mode (top left), stimulator, recorder, calibration, and filter settings tabs (middle left), buttons for connecting to the SRD, sending/receiving settings to/from SRD, and saving/loading settings from a file saved to the PC (bottom left), options for saving waveforms to file (top right), options for viewing live or previous waveform files (middle right), and graphs of all 12 available channels with sinusoidal waveforms shown for demonstration purposes (bottom right). Recorder settings include the trace count (number of traces captured during each recording epoch used in automatic mode), epoch interval, trace length, sampling rate, and active channels. **b** Stimulator tab settings include amplitudes, durations, inter-phase interval, frequency, and stimulation delay (relative to start of recording). **c** Calibration tab settings include ADC calibration and stimulation calibration gain and offset (assumes linear calibration). **d** Filter tab settings include − 3 dB cutoff frequencies for f_L_, f_H_, and f_D_

The SRD’s firmware handles all hardware control and processing that takes place on the SRD’s PCB. Firmware for the SRD was designed using PSoC Creator 3.0 (Cypress Semiconductor). Firmware code was written using C and compiled using the PSoC Creator’s integrated compiler. All stimulation and recording settings are configurable using a custom GUI with additional ability to view and save signals streaming from the SRD (Fig. 8).

Six SRD operating modes are available: Stand-By, Automatic, Continuous, External Stimulation, External Recording, and Look-Ahead External. Stand-By places the SRD in a quiescent mode whereby no stimulation, recording, or wireless outbound data transmissions are occurring and several of the PSoC components are switched to a low power operating state to conserve energy. Automatic mode stimulates and records on a user pre-programmed schedule. All signals are buffered on-chip and then sent to the PC, looping until manually stopped by the user. The user can set an active and sleep period whereby the SRD is either actively stimulating/recording or sleeping until the next period of activity. Continuous mode samples signals from the amplifiers and immediately sends signal data to the PC for viewing in a streaming fashion, but is limited to a single channel. External Stimulation mode is used to trigger the SRD’s stimulation function using an external source. By default, the on-board IR sensor is used as the trigger input source, but auxiliary inputs could be programmed for trigger inputs within the firmware. When an IR pulse is detected, the SRD delivers a single stimulation event according to settings defined in the GUI. Similarly, External Recording mode is used to trigger the SRD to record a single trace via an external source with the on-board IR sensor as the default trigger input source. The Look-Ahead External mode continually buffers signals from the amplifiers and sends signal data to the PC only when an external trigger is detected, which is useful for experiments in which a behavioral response (e.g. bar press by the rat) is used to trigger recording.

### SRD system summary

Table 2 summarizes the system that includes enclosure and PCB size, and specifications of the recorder, stimulator, and power subsystems. A key design requirement for the SRD was cost and accessibility to users. Totaling the SRD circuit components, backpack, wearable vest, and PCB manufacturing services yields a cost of less than $500 USD. Note that the specifications reflect our intended applications in intra- and interhemispheric connectivity studies. For example, the stimulator frequency range of 0.2–5.0 Hz will be used for repetitive, low frequency ICMS in rat SI cortex, which has been shown to induce expansion of receptive fields around the stimulation site to include adjacent cortical territory [33] as well increase evoked responses due to ICMS at the homotopic site in the opposite hemisphere and induce ipsilateral input that was not present prior to ICMS [17].

**Table 2:** SRD specifications

System	Parameter	Specification
Entire System	PCB Size Enclosure	48.0 × 27.0 mm
		53.5 × 33.5 × 16.9 mm
	Weight	Enclosure: 14.8 g
		PCB: 7.1 g
		Battery: 17.1 g
		EIB: 0.2 g
		Connector/wire: 0.7 g
	Battery Life	37 h (during standard continuous experimental operation)
Recorder	Sample Rate	1–15 ksps/channel
	Resolution	16 bit
	Channels	1 or 2 (12 available)
	Filter Bandwidths	0.1–500 Hz (high pass)
		100 − 20 k Hz (low pass)
		< 1–1655 Hz (digital high pass)
	Noise Floor	± 5 μV (inputs grounded)
Stimulator	Waveform Shapes	biphasic, monophasic, pseudophasic
	Phase Duration	0.5–5.0 ms
	Inter-Phase Interval	0.1–5.0 ms
	Frequency	0.2–5.0 Hz
	Amplitude	± 255 μA (1-μA increments)
	Compliance Voltage	± 10 V
Power	Voltage Input	3.7–5.1 V
	Average Current	~ 27 mA

### Animal testing and experimentation

Animals and Animal Preparation–The SRD was tested in adult female Sprague Dawley rats (Harlan) weighing 200–300 g. Rats were single-housed in standard cages with ad lib. access to water and rat chow in an animal room (12/12 h light/dark cycle) within the Laboraroty Animal Care Unit facilities at the University of Tennessee Health Science Center (UTHSC).

Stimulation waveform testing was examined in two rats using Ketamine/Xylazine (87/13 mg/kg, i.m.) and supplemented hourly (10% of initial dose) or sooner if needed throughout testing to maintain areflexia. A water-circulating heating pad was used to maintain body temperature between 36.5 °C and 38.0 °C throughout the experiment. Under aseptic conditions, rats were placed in a stereotaxic apparatus, the skull over SI cortex was exposed, and a craniotomy (2–3 mm diameter) was performed bilaterally at 0.3 mm posterior and 3.5 mm lateral to bregma. Four additional holes were drilled in the posterior skull for grounding screws and support (Fig. 7). A platinum-iridium (Pt/Ir) microelectrode (Microprobes) with impedance of 100 kΩ at 1 kHz was used for stimulation waveform testing. The microelectrode was attached to a microdrive and inserted through the dura into SI cortex at a depth between 900 and 1,400 μm. Stimulation current and voltage drop across the stimulating electrode were captured with a digital oscilloscope (Rigol DS2072) and precision current meter (μCurrent).

Repetitive ICMS testing was examined in two rats. Anesthesia was induced with isoflurane (5%, 1 L/min O_2_, 3–5 min) and maintained (1.5–2.5%, 0.2–0.4 L O_2_) throughout surgery. Craniotomies were performed under aseptic conditions as described above. A Pt/Ir microelectrode (1 MΩ at 1 kHz) was attached to a microdrive, inserted through the dura into SI cortex at a depth between 900 and 1,400 μm and used to record receptive fields of forelimb neurons evoked by mechanical stimulation [31]. When a receptive field of interest was identified, the electrode shaft was fixed to the skull with dental cement (Fig. 7b). After hardening, the electrode guide tube was retracted leaving the electrode lead wire and guide post exposed, the latter of which was snipped off. A second Pt/Ir electrode (10 kΩ at 1 kHz) attached to a microdrive was inserted into opposite SI cortex to identify a homotopic forelimb receptive field. Upon identification, single-pulse ICMS (pulse duration, 1-ms; amplitude, 10–50 μA; interval, 1 Hz) was delivered to the electrode to evoke a response in the opposite SI cortex to confirm connectivity. The electrode was then fixed in place.

The EIB was lowered into position over the implant sites (Fig. 7). Electrode lead wires were placed into through-holes on the EIB and secured using friction-fit gold-plated pins (Neuralynx); excess lead wire was trimmed. A dental cement cap was formed over all screws, lead wires, and around the EIB. The SRD was connected to the newly formed headstage using a cable constructed with 18 stranded, insulated wires (34-gauge) and two female connectors (A9847–801, Omnetics) and interhemispheric connectivity was reconfirmed using ICMS.

Immediately following surgery, rats were given an antibiotic (penicillin G, 0.05 mL, i.m.) and analgesic (buprenorphine HCl, 0.03 mg/kg, i.m.); an antibiotic ointment was applied around the incision. Rats were monitored until regaining consciousness, and returned to the vivarium for recovery. The surgical site was visually inspected daily for signs of inflammation or infection. Anesthesetics, analgesics, and antibiotics used were consistent with previous studies and conformed to animal care policies of UTHSC.

Following recovery (~ 2 days), the SRD was attached and all experimental parameters were sent to the SRD via the GUI. Repetitive single-pulse ICMS (biphasic, 1 ms, 100 μA, 1 Hz) was delivered to the stimulating electrode in SI cortex for 0.5–3 h. The pulse duration and amplitude were chosen to reflect upper limits used in our intended application for interhemispheric stimulation. Cortico-cortical evoked responses were continuously collected from the recording electrode in the opposite SI with filter settings of f_L_ = 100 Hz, f_H_ = 1 kHz, and f_D_ = 318 Hz, sampling rate of 15 ksps, and sampling duration of 100-ms per ICMS pulse. In-vivo testing of the SRD’s ability to synchronize with external stimulation system was conducted with the rat under isoflurane anesthesia. Evoked responses were recorded in forelimb SI cortex following electrical stimulation of the contralateral forelimb skin surface with a pair of silver wire electrodes (100 μA maximum intensity, 1-ms pulse, 1 Hz). Contralateral hindlimb and ipsilateral forelimb were also stimulated as controls. The external IR sync device was connected to the peripheral stimulator system and positioned 12-in above the SRD. One-way repeated measures analysis of variance (ANOVA) with Tukey post-hoc tests were used to test for statistically significant differences in 10 consecutive responses at differing time points (α = 0.05).

Following testing, rats were euthanized by administering a lethal dose of Nembutal (100 mg/kg, i.p.) and transcardially perfused with 4% paraformaldehyde. Excised brains were fixed in 4% paraformaldehyde, cut in 100-μm thick coronal sections, and stained with cytochrome oxidase to visualize recording and stimulation sites [31].

## Data Availability

Design files for the circuit schematic, circuit PCB, SRD enclosure [34], SRD firmware [35], SRD GUI [36], and external IR sync pulse emitter PCB [37] are available in online repositories.

## Abbreviations

ADC: Analog-to-digital converter
AMUX: Analog multiplexer
ANOVA: Analysis of variance
BT: Bluetooth
EIB: Electrode interface board
f_D_: Digital high-pass filter frequency
f_H_: Upper cutoff frequency
f_L_: Lower cutoff frequency
GUI: Graphical user interface
I/O: Input/output
ICMS: Intracortical microstimulation
IDAC: Current-mode digital-to-analog converter
IR: Infrared
LDO: Low-dropout
LED: Light-emitting diode
LiPo: Lithium polymer
MCU: Microcontroller unit
MOSFET: Metal-oxide-semiconductor field-effect transistor
PC: Personal computer
PCB: Printed circuit board
PSoC: Programmable system on a chip
RMS: Root mean square
SI: Primary somatosensory cortex
SPI: Serial peripheral interface
SRD: Stimulation-and-recording device
UART: Universal asynchronous receiver/transmitter
USB: Universal serial bus
UTHSC: University of Tennessee Health Science Center
ZIF: Zero-insertion force
